# Encountering Peritoneo-Cutaneous Perforators in Microsurgical DIEP Flap Breast Reconstruction

**DOI:** 10.1055/a-2006-0747

**Published:** 2023-03-28

**Authors:** Duncan Loi, Justin L. Easton, Warren M. Rozen

**Affiliations:** 1 Department Surgery, Peninsula Clinical School, Central Clinical School, Faculty of Medicine, Monash University, Frankston, Victoria, Australia; 2 Monash University Plastic and Reconstructive Surgery Group (Peninsula Clinical School), Peninsula Health, Frankston, Victoria, Australia

**Keywords:** microsurgery, breast reconstruction, anatomy

## Abstract

The vascular anatomy of the deep inferior epigastric artery perforator (DIEP) flap has been well studied in the planning for autologous breast reconstruction. Preoperative imaging with computed tomography angiography (CTA) provides accurate assessment of this vascular anatomy, which varies widely across patients. Several papers to date have described their encounter with an anomalous “epiperitoneal” or “peritoneo-cutaneous” perforator during flap harvest, a perforator that pierces the posterior rectus sheath from a peritoneal origin, to traverse rectus abdominis and supply the DIEP flap integument. In the course of over 3,000 CTA assessments of the vascular anatomy of the abdominal wall, we have encountered dominant peritoneo-cutaneous perforators in 1% of cases, and smaller perforators seen in many more cases, approaching 5% of cases. With increasing sensitivity of imaging, we also describe a unique case of multiple large bilateral peritoneo-cutaneous perforators, and present these findings in the context of DIEP flap harvest. It is critical to recognize these peritoneo-cutaneous perforators preoperatively to avoid mistaking them for a DIEP during the raising of a DIEP flap. The routine use of preoperative CTA enables the safe identification of individual vascular anatomy, including significant peritoneo-cutaneous perforators.

## Introduction

As a mainstay of autologous breast reconstruction, the deep inferior epigastric artery (DIEA) perforator (DIEP) flap and its vascular anatomy have been well studied. The DIEA, originating in the majority of cases from the external iliac artery, provides the blood supply to the skin and soft tissues of the lower abdominal wall. 1 Preoperative imaging with computed tomography angiography (CTA) has become routine practice in many centers for DIEP flap planning, and provides accurate assessment of the vascular anatomy, which varies widely across patients. This has allowed safer intraoperative dissection, and translated to reduced operative time and improved overall flap outcomes. 2 3 From its origin on the external iliac artery, the DIEA courses superomedially toward the lateral edge of the rectus sheath before approaching the deep aspect of the rectus abdominis. At this point, the path of the artery can be understood in multiple segments: the DIEA course deep to the rectus abdominis muscle, the intramuscular course of the DIEA, the intramuscular course of the DIEA perforator, the perifascial course of the perforator, and the subcutaneous course of the perforator.

Several papers to date have described their encounter with an anomalous “epiperitoneal” or “peritoneo-cutaneous” perforator during the raising of a DIEP flap for breast reconstruction, 4 5 6 a perforator that pierces the posterior rectus sheath from a peritoneal origin, to traverse rectus abdominis and supply the DIEP flap integument. With the increasing sensitivity of imaging, imaging of this anatomy has improved, and we report a further variant on this aberrant vascular anatomy, that of multiple peritoneo-cutaneous perforators providing supply to the abdominal wall, and present this in the context of DIEP flap harvest.

## Idea

Lasso et al 4 described the presence of a single large medial periumbilical perforator that was found to pierce deep to the posterior fascia, and found to augment the blood flow to their DIEP flap. Whitaker et al 6 further investigated this anomaly in a series of cadaveric dissections and CTA analyses, confirming the presence of a significantly sized peritoneo-cutaneous perforator supplying and draining the abdominal wall in approximately 1% of patients in their study. These perforators were seen to have no communication with the DIEA. These studies identified single large perforators in their cases. The current study comprises a case report and context of this case within our clinical experience. All CTAs described herein were performed at a single institution, using a standardized 128-slice CTA scanner and single protocol for scanning. Each CTA scan was reported by a single person, using Horos (The Horos Project, Nimble Co LLC Purview, Annapolis, MD) to map out each perforator incorporated within the abdominally based flaps planned. The data presented comprises a consecutive series, with no exclusions.

Institutional human research ethics committee approval was achieved (approval numbers 2006.038; 2006.231 and HREC86700PH-2022), and full informed patient consent was undertaken. We thus describe the case of a 48-year-old female who underwent abdominal wall CTA as part of routine preoperative workup for DIEP flap breast reconstruction. Preoperative analysis of CTA images revealed the presence of multiple bilateral peritoneo-cutaneous perforators, a unique finding, as described above. In the course of over 3,000 CTA assessments of the vascular anatomy of the abdominal wall, we have encountered dominant peritoneo-cutaneous perforators in 1% of cases, matching the findings of Whitaker et al. However, smaller such perforators have been seen in many more cases than this, approaching 5% of cases. These perforators share some anatomical features, highlighted in Table 1 , highlighting that these are largely periumbilical and medial row in location. In all cases, there appeared to be no effect on DIEA perforators, with adequate DIEA perforators able to be selected and utilized for flap harvest.

**Table 1:** Anatomical features of peritoneo-cutaneous perforators in 3,000 breast reconstruction cases

Anatomical feature	Number of cases
Size > 1 mm	30 (1% incidence)
Size > 0.5 mm	150 (5% incidence)
Dominant perforator medial row	28/30 (93%)
Dominant perforator lateral row	2/30 (7%)
Dominant perforator located periumbilically	28/30 (93%)

**Fig. 1:**
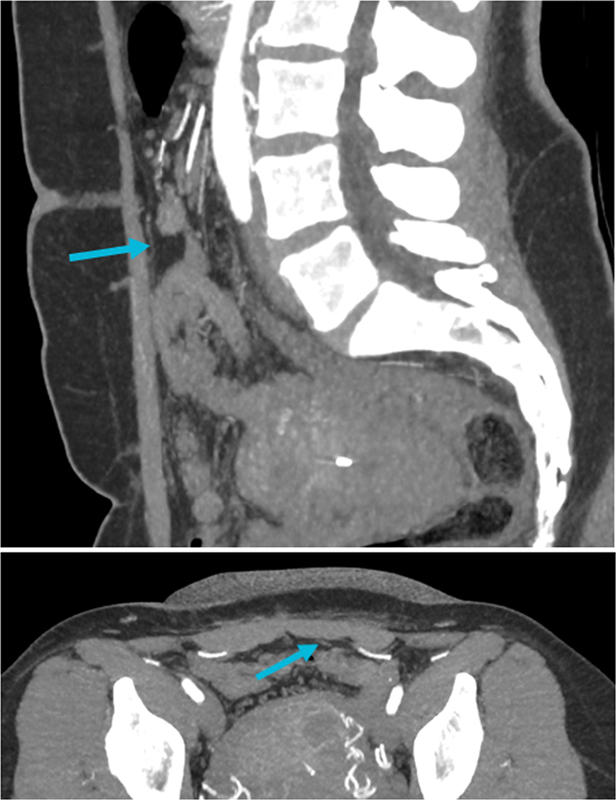
Computed tomographic angiogram (CTA) axial slices demonstrating a peritoneo-cutaneous perforator (blue arrows).

**Fig. 2:**
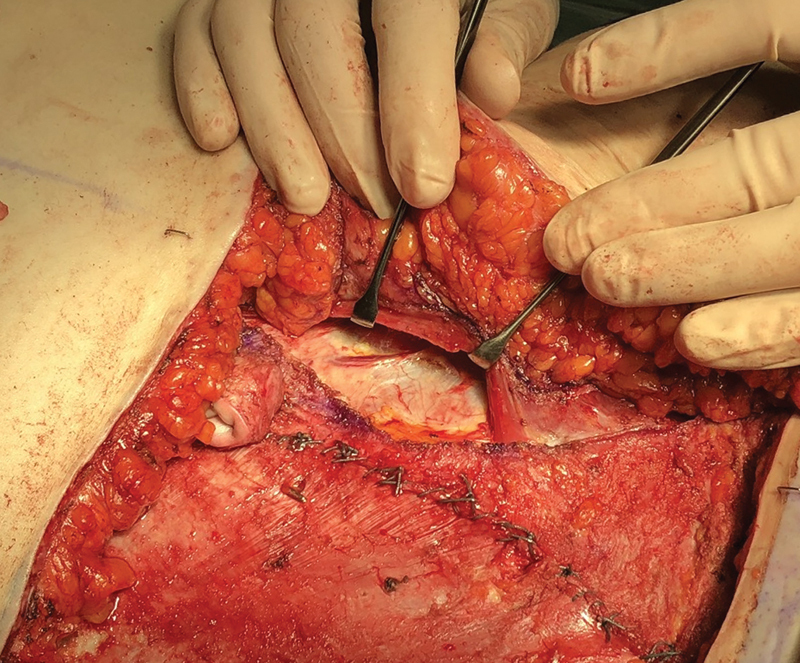
Intraoperative photograph demonstrating three large peritoneo-cutaneous perforators, all over 1 mm in diameter.

In our case of multiple bilateral peritoneo-cutaneous perforators, it was found that all perforators identified were periumbilical and shared no anastomoses with the DIEA perforator system ( Fig. 1 ). The DIEAs originated on either side from the obturator arteries, but otherwise followed a standard course, giving off large medial row perforators that appeared suitable for a perforator flap. These peritoneo-cutaneous perforators identified on preoperative imaging were confirmed intraoperatively ( Fig. 2 ). During initial flap dissection and raising, multiple perforators supplying the lower abdominal wall bilaterally were seen to originate deep to the posterior rectus sheath. The course of the largest of these perforators was dissected, and was confirmed to traverse the extraperitoneal fat, posterior rectus sheath, rectus abdominis muscle, and subcutaneous tissue, and had no direct communication with the DIEA. Ultimately, bilateral DIEP flaps were raised bilaterally, and all identified peritoneo-cutaneous perforators were clipped and ligated, with successful flap transfer and no complications.

## Discussion

Peritoneo-cutaneous perforators are an uncommon anatomical variant in the arterial supply of the abdominal wall. Significantly sized peritoneo-cutaneous perforators are thought to occur in about 1% of patients, 6 though the presence of smaller perforators may be more frequent than otherwise thought. In our case, however, there were in fact multiple large peritoneo-cutaneous perforators bilaterally, several of which were over 1 mm in diameter. It is critical to recognize these peritoneo-cutaneous perforators preoperatively to avoid mistaking them for a DIEA perforator during the raising of a DIEP flap, should they be present. Alternatively, they may also be used as a second perforator to augment blood flow to a DIEP flap. 4

Innumerous small peritoneal branches from the entire DIEA provide supply to the parietal peritoneum concordant with its cutaneous territory. 7 This is the anatomical basis for the composite musculo-peritoneal free flap. 8 However, Whitaker et al demonstrated these peritoneo-cutaneous perforators to be separate to the DIEA system by directly injecting contrast mixture in the perforators found in their cadaveric study. Peritoneo-cutaneous perforators may be considered an example of abnormal vasculogenesis. While it has been long held that the umbilicus receives supply from intra-abdominal vessels, it is conceivable that the periumbilical perforators that originate intra-abdominally represent a developmental abnormality whereby umbilical vessels have developed a more extensive pattern of supply. 9 10 These are vessels that run within the ligamentum teres and the median umbilical ligament, which normally contribute and anastomose with the periumbilical vascular plexus.

Preoperative CTA has already been proven to be a useful adjunct by reducing operative times and allowing safer intraoperative flap dissection. 2 3 The routine use of preoperative CTA enables the safe identification of individual vascular anatomy and indeed the presence or absence of any significant peritoneo-cutaneous perforators.
